# Effects of Arm Crossing on Spatial Perspective-Taking

**DOI:** 10.1371/journal.pone.0095748

**Published:** 2014-04-21

**Authors:** Tiziano Furlanetto, Alberto Gallace, Caterina Ansuini, Cristina Becchio

**Affiliations:** 1 Centre for Cognitive Science, Department of Psychology, Università degli Studi di Torino, Torino, Italy; 2 Department of Psychology, Università degli Studi di Milano Bicocca, Milano, Italy; 3 Department of Robotics, Brain and Cognitive Science, Fondazione Istituto Italiano di Tecnologia, Genova, Italy; University G. d’Annunzio, Italy

## Abstract

Human social interactions often require people to take a different perspective than their own. Although much research has been done on egocentric spatial representation in a solo context, little is known about how space is mapped in relation to other bodies. Here we used a spatial perspective-taking paradigm to investigate whether observing a person holding his arms crossed over the body midline has an impact on the encoding of left/right and front/back spatial relations from that person’s perspective. In three experiments, we compared performance in a task in which spatial judgments were made from the perspective of the participant or from that of a co-experimenter. Depending on the experimental condition, the participant’s and the co-experimenter’s arms were either crossed or not crossed over the midline. Our results showed that crossing the arms had a specific effect on spatial judgments based on a first-person perspective. More specifically, the responses corresponding to the dominant hand side were slower in the crossed than in the uncrossed arms condition. Crucially, a similar effect was also found when the participants adopted the perspective of a person holding his arms crossed, but not when the other person’s arms were held in an unusual but uncrossed posture. Taken together these findings indicate that egocentric space and altercentric space are similarly coded in neurocognitive maps structured with respect to specific body segments.

## Introduction

Results from neurophysiology, neuropsychology, and psychophysics converge in showing that the egocentric representation of the space surrounding the body is structured with respect to specific body parts, such as the hands or the face, and can plastically change depending on bodily action possibilities [1], [2], [3]. For instance, it is already well known that the active use of a tool extending reachable space can cause the remapping of far space as near space [4] and that peripersonal space can be shifted to include the position of artificial body parts (e.g., [5], [6]).

A far less explored issue is that of how space is remapped in relation to other bodies [7], [8], [9]. People are inherently social beings and often find themselves in situations that require them to overcome their own position in space to adopt another person’s spatial perspective. When asking another person where an object is located, for example, people typically favor the other person’s spatial perspective over their own and tend to answer from that person’s viewpoint (e.g., “on your left”; [10]). Similarly, they may adopt the spatial perspective of another person who is in the position to act on objects [11], even more so when the person’s behavioral intention is ambiguous and the need for action understanding is therefore increased [12].

Effects of spatial perspective-taking are not limited to the type of linguistic descriptors used (e.g., “on your left” rather than “on my right”), but reflect a spatial remapping of objects and locations with reference to the other person’s body (i.e., altercentric frame of reference [13]). In a recent study, brain damaged patients affected by left egocentric spatial neglect – a failure in attending and reporting stimuli on the contralesional side of body-centered space – were asked to describe different arrays of objects either from their own perspective or from that of another person seated in front of them [13]. Items presented on the affected side of space and omitted when report was required from the first-person perspective could be recovered when patients assumed the other person’s perspective, suggesting that object location had been remapped within a preserved altercentric frame of reference.

Taken together these findings suggest that, similarly to spatial recalibration induced by tool use, perspective-taking may involve a ‘social recalibration’ of spatial representations [9]. People adopting another person’s spatial perspective remap object locations so as to anchor the description of spatial relations to the other person’s point of view rather than to their own. The extent to which they ‘embody’ the other person’s point of view and actually transport themselves into the other person’s body posture remains, however, unclear.

Studies investigating imagined transformations of whole body perspective, as indexed by the own body transformation (OBT) task, consistently show that laterality judgments regarding the handedness of a schematic figure are faster and more accurate when the figure shares the same spatial orientation as the participant (i.e., orientation effect; [14], [15], [16], [17], [18], [19]). Along the same lines, posture congruency, (i.e., congruency between one’s own body posture and that of the other person), and movement congruency, (i.e., congruency between the participant’s body posture and the direction of mental self-rotation necessary to align perspectives), have been shown to affect spatial judgments regarding object locations [20], [21]. For example, it has been demonstrated that judging whether an object is on the left or right of someone becomes easier when one’s own body posture matches that of the other person, suggesting that remapping of left/right relations within an altercentric frame of reference is affected by posture congruency [21], [22]. Critically, however, whether altercentric remapping is related to the other person’s body as a whole or to specific body parts, has not yet been determined. Moreover, no study has so far investigated whether the other person’s body/limb posture may have a specific effect on the processing of spatial relations (e.g., selectively affecting spatial relations in the left-right dimension but not in the front-back dimension).

To address these issues, in the current study we applied a manipulation that is frequently used when studying peripersonal space: crossing the arms over the body midline. By changing the spatial correspondence of body sense information to distal locations, this manipulation has been shown to lead to measurable changes in spatial compatibility (e.g., [23]), spatial attention (e.g., [24]), and to a decrement in the ability to detect tactile stimuli [25]. Furthermore, there is evidence that crossing the hands reduces the orientation effect in own body transformation tasks [26], modulates the integration of multisensory information in peripersonal space [27], [28], and reduces the intensity of pain evoked by noxious stimulation of the hand [29], [30].

In the present study, we aimed to investigate whether observing a person holding his arms crossed over the midline influences encoding of left/right and front/back spatial relations from that person’s perspective. In three experiments, we compared performance in a task in which spatial judgments were made from the perspective of the participant or from that of a co-experimenter. Depending on the experimental condition, the participant’s and the co-experimenter’s arms were either crossed or not crossed over the body midline. We predicted that: i) holding the right hand in the left space and the left hand in the right space would have a specific impact on left/right spatial judgments from a first-person perspective (Experiment 1); ii) observing a person holding his/her arms crossed over the midline would exert similar effects on spatial judgments from a third-person perspective (Experiment 2); iii) no such specific effects on left/right spatial judgments from a third-person perspective would be apparent when taking the perspective of a person with his arms uncrossed in an unusual posture. This would indicate that when taking another’s person perspective, participants map object locations in relation to the specific limb posture, rather than in relation to a mere altercentric bodily point or to the other person’s body as a whole.

## Experiment 1

Experiment 1 was designed to test the effects of crossing the arms over the midline on spatial judgments from a first-person perspective. The participants were presented with different arrays of everyday objects and asked to answer simple questions regarding the position of an object in relation to that of another object (e.g., “In relation to the mug, where is the alarm clock?”) while holding their arms either crossed or not crossed over their body midline. Questions included front/back judgments and left/right judgments.

Studies investigating access to objects in the horizontal plane indicate that reaction times to identify objects at specific locations are faster for the front-back axis then for the left-right axis (e.g., [31]). Moreover, there is evidence of asymmetries in processing matches involving the terms “right” and “left”. In particular, right-handers have been shown to be faster in processing right relations (e.g., [32]).This might be due to the effect of acquired experience in interacting with the physical environment more efficiently on their dominant side and less efficiently on their non-dominant side [33]. Similar biases in processing information to the right of the participants’ own bodies have been found in spatial choice-reaction tasks. Responses to visual stimuli are faster to stimuli in the visual field corresponding to the dominant hand [34] and the Simon effect (i.e., faster responding when irrelevant stimulus location corresponds with response location than when it does not) is greater in the right visual field for right-handers and the left visual field for left-handers [35], [36]. In line with this literature, we predicted two effects. First, we expected that response latencies would become overall longer in the crossed posture compared to the uncrossed posture, reflecting the increased processing costs induced by the adoption of an unusual posture. This effect should be observed for both front/back judgments and left/right judgments, with judgments for front and back spatial relations being overall faster than judgments for left and right relations. Second, as a consequence of the mismatch between the codes used to describe the relative position of the hand and the side of the body with which the hand is connected [37], we hypothesized that crossing the arms would exert a specific effect on judgments related to the left-right axis. In spatial choice-reaction tasks, crossed-hands manipulation has been shown to bring about a reversal of the asymmetry associated with handedness [35]. Similarly, we expected that crossing the arms would slow down judgments regarding right positions, reducing the magnitude of the effect of hand dominance or eliminating it altogether.

### Methods

#### Participants

Thirty undergraduate students (15 females; mean age: 23.80±3.54, range 20–38 years) from the University of Turin volunteered to take part in the experiment. All had normal or corrected-to-normal vision, were naïve to the purpose of the stud and right handed. Handedness was determined through the Edinburgh handedness questionnaire [38]. Written informed consent was obtained from all participants. The study was performed in accordance with the ethical standards laid down in the 1991 Declaration of Helsinki and was approved by the Ethics Committee of the University of Turin.

#### Materials and apparatus

The participants were seated at a table (100×100 cm) centered on their sagittal midline. Sixteen objects of common use (see Table 1) were placed on the table within an 80×80 cm array (four rows by four columns). The participants were required to answer simple questions regarding the position of an object in relation to that of another object (e.g., “In relation to the mug, where is the alarm clock?”). They were instructed to answer (“front”, “back”, “left”, “right”), after the end of each question as quickly and accurately as possible. The questions were recorded in a male voice and played to the participants through stereo headphones. Each question lasted 6.95 seconds. The participants’ vocal reaction times (RTs) were recorded. Response accuracy was recorded manually by the experimenter, sitting behind the participant.

**Table 1:** Stimuli used in the study.

N#	Name^a^
**1**	Alarm clock
**2**	Can
**3**	Cellphone
**4**	Funnel
**5**	Glove
**6**	Hairbrush
**7**	Highlighter
**8**	Mug
**9**	Pin box
**10**	Playing card deck
**11**	Safety lock
**12**	Scissors
**13**	Sponge
**14**	Stapler
**15**	Sunglasses
**16**	Wrench

a Objects are listed in alphabetical order.

#### Design and procedure

**Figure 1:**
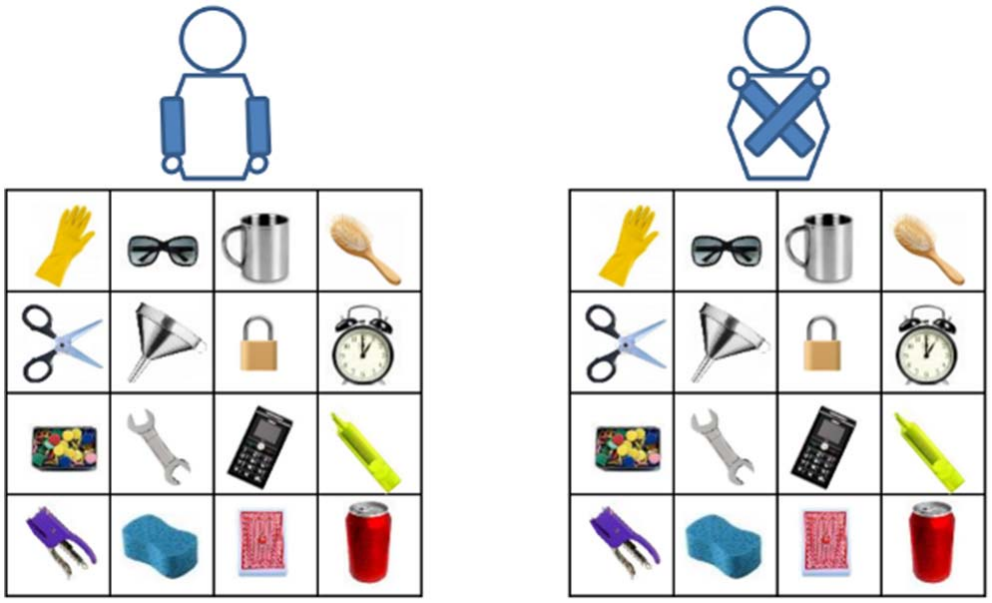
Schematic representation of the set-up and stimuli of Experiment 1. Left and right panels represent uncrossed and crossed arm posture conditions, respectively. Please note that the objects shown and their displacement are illustrative and do not reflect those actually used.

During the experiment, the participants were asked to keep their arms either uncrossed or crossed. In the uncrossed position, they kept their arms uncrossed with their hands on the armrest of the chair (see Figure 1); in the crossed position they held their arms crossed over the midline so that the left hand was placed on the right shoulder and the right hand on the left shoulder. Uncrossed and crossed position trials were run in two separate blocks. The participants answered 32 questions in each block, 16 requiring judgments of front and back relations and 16 requiring judgments of left and right relations. This allowed presentation of each object twice as the first object and twice as the second object. After completion of the first block, the array was rotated by 90° clockwise with respect to the participant’s position. To control for object-centered spatial processing (i.e., encoding of spatial locations in accordance with a reference object [39], [40]), front/back and left/right questions in each block of trials were presented in a randomized order. The order of the blocks was counterbalanced across participants. The experiment lasted approximately 15 minutes.

#### Analysis

Data analysis focused on RTs. Trials in which participants made an error were discarded from the RT analysis (3.12%). In addition, individual trials were removed if responses were made less than 150 ms after the end of the question (2.50%) or in excess of two standard deviations of the participant’s mean reaction time (3.59%). RTs were initially analyzed using a repeated measures ANOVA with *arm posture* (uncrossed vs. crossed) and *response axis* (front/back vs. left/right) as within-subjects factors. Additionally, in order to test the hypothesis that crossing the arms would specifically affect judgments concerning left and right locations, but not judgments concerning front and back locations, in a second analysis, repeated measures ANOVAs were computed on RTs separately for left/right and front/back judgments. *Arm posture* (uncrossed vs. crossed) and *response* (left vs. right; front vs. back) were used as within-subjects factors. The alpha level was set at 0.05. Where multiple t-tests were used, correction for multiple comparisons was made by dividing the alpha level by the number of comparisons.

### Results

The ANOVA analysis on RTs revealed a main effect of *arm position* (*F*(1, 29) = 5.779, *p* = .023, η^2^ = .166), with RTs being slower in the crossed (*M* = 515,9 ms) than in the uncrossed posture (*M* = 466,9 ms). Furthermore, there was a main effect of *response axis* (*F*(1, 29) = 10.304, *p* = .003, η^2^ = .262), reflecting slower responses for judgments of left and right relations (*M* = 502 ms) than for judgments of front and back relations (*M* = 480,7 ms). The interaction *arm posture* by *response axis* was not significant (*F*(1,29) = .049, *p* = .826, η^2^ = .002).

#### Analysis of RTs for left/right judgments

**Figure 2:**
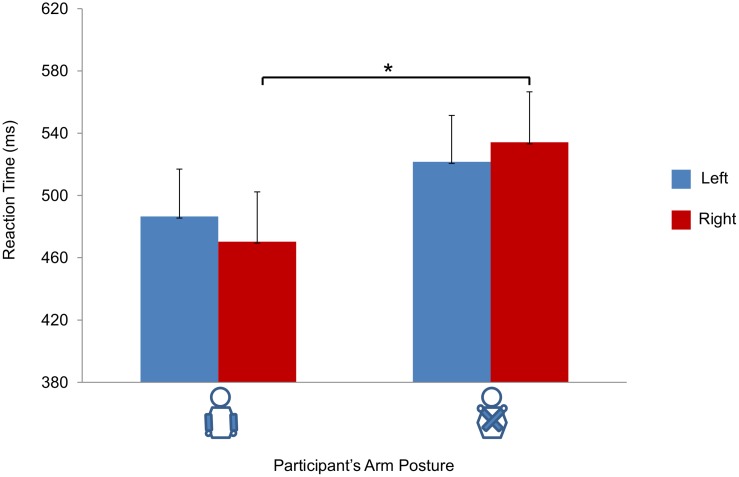
Results of Experiment 1 for left vs. right judgments. Graphical representation of the interaction *Response* (left vs. right) by *Arm Posture* (uncrossed vs. crossed) on Reaction Times. Bars represent standard errors of the means. Asterisks indicate significance for the main contrasts of interest (*p*<05).

The ANOVA revealed a main effect of *arm posture* (*F*(1, 29) = 5.059, *p* = .032, η^2^ = .149), with RTs being slower in the crossed (*M* = 527,9 ms) than in the uncrossed posture (*M* = 478,4 ms). The main effect of *response* was not significant (*F*(1, 29) = .65, *p* = .800, η^2^ = .002), but there was a significant *arm posture* by *response* interaction effect (*F*(1, 29) = 4.351, *p* = .046, η^2^ = .130). A paired t-test showed that right responses were faster in the uncrossed posture (M = 470,4 ms) than in the crossed posture (M = 534,2 ms) (*t = −*3.004, *p* = .005). In contrast, no significant difference was found when comparing left responses in the uncrossed posture (M = 486,5 ms) and in the crossed posture (M = 521,6 ms) (*t = −*1.420, *p* = .166; see Figure 2). No other significant differences were observed.

#### Analysis of RTs for front/back judgments

The ANOVA revealed a main effect of *arm posture* (*F*(1, 29) = 5.249, *p* = .029, η^2^ = .153), reflecting faster RTs when participants’ arms were uncrossed. The main effect of *response* and the *arm posture* by *response* interaction effect were not significant (all *Fs<*.578, *p>*.453*).*

In sum, these findings suggest that over and above a general effect reflecting the increased processing costs induced by the adoption of an unusual posture, crossing the arms specifically influenced left/right judgments, with right responses, but not left responses, being faster in the uncrossed than in the crossed posture. In contrast, as revealed by the lack of interaction between *arm posture* and *response*, front and back responses were equally affected by the crossed-hands manipulation.

## Experiment 2

The findings of Experiment 1 indicate that arm posture exerts a specific influence on spatial judgments from a first-person perspective. Experiment 2 was designed to investigate whether observing a person holding his arms crossed over the midline would have a similar impact on the encoding of spatial relations from that person’s perspective (i.e., third-person perspective). Studies investigating spatial perspective-taking consistently report that third-perspective judgments of front and back relations are faster than judgments of left and right (e.g., [22]). As for first-person perspective, we therefore expected RTs to be faster for the antero-posterior axis than for the left-right axis. If overall judgments were also affected by the other person’s body posture, this would suggest that altercentric remapping relates to the other person’s arm posture.

### Methods

#### Participants

Twenty-one undergraduate students (10 females; mean age: 22.81±3.03, range 20–32 years) from the University of Turin volunteered to take part in the experiment. All had normal or corrected-to-normal vision, were right handed, and naïve to the purpose of the study. None of the participants who took part in Experiment 1 participated in Experiment 2. The sample size was determined on the smallest effect in Experiment 1 (namely, the prevalence of uncrossed vs. crossed responses for right answers) so as to ensure a 95% power of rejecting the null hypothesis.

#### Materials and apparatus

The materials and apparatus were the same as in Experiment 1 with the following exceptions: during the experiment, a male co-experimenter was seated at the table, to the right of the participant, and looked at the objects with an angular disparity of approximately 100° with respect to the participant’s view. As in Experiment 1, the participants were required to answer simple questions regarding the position of an object in relation to that of another object. However, instead of answering from their own point of view, they were asked to answer from the perspective of the co-experimenter (i.e., third-person perspective).

#### Design and procedure

**Figure 3:**
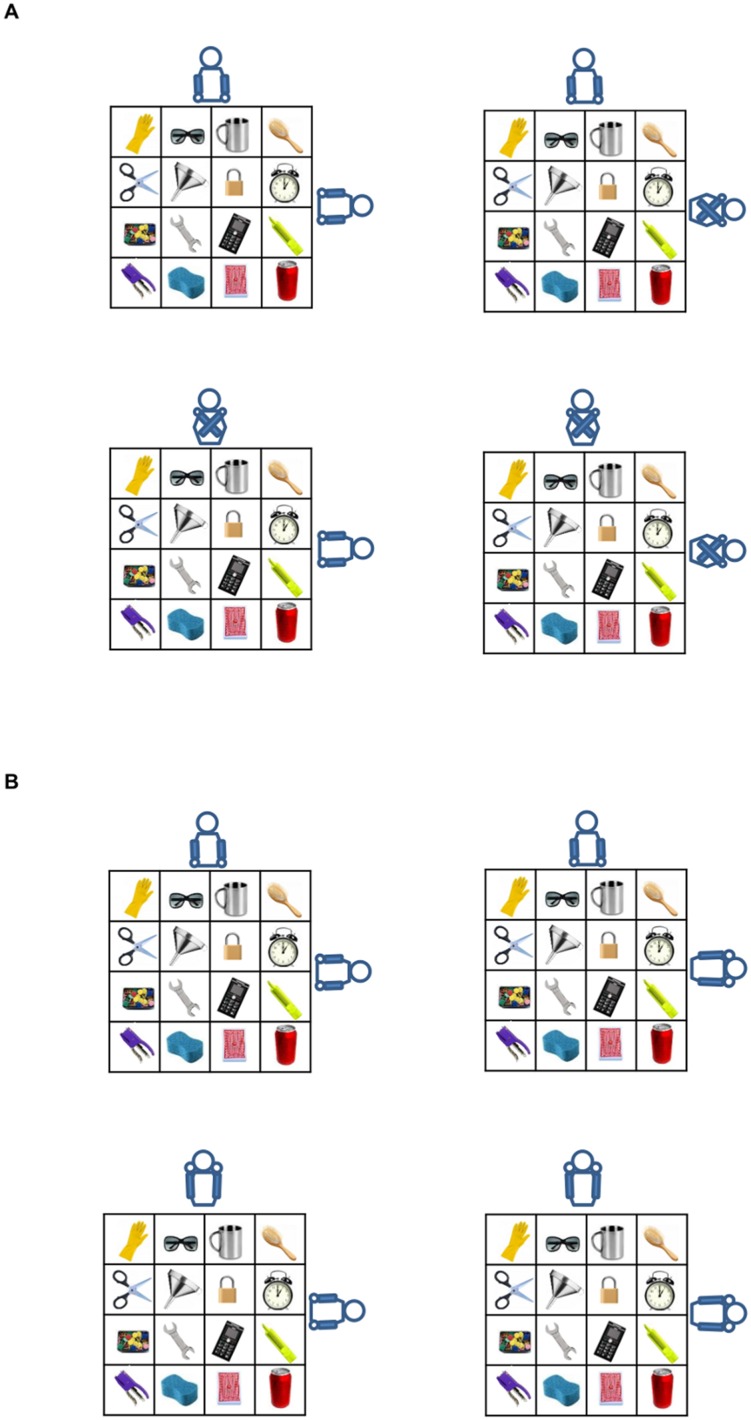
Schematic representation of the set-up and stimuli adopted in Experiments 2 and 3. In Panel A, the four experimental conditions of Experiment 2: participant uncrossed/co-experimenter uncrossed; participant uncrossed/co-experimenter crossed; participant crossed/co-experimenter uncrossed, and participant crossed/co-experimenter crossed. In Panel B, the four experimental conditions of Experiment 3: participant usual/co-experimenter usual posture; participant usual/co-experimenter unusual posture; participant unusual/co-experimenter usual posture, and participant unusual/co-experimenter unusual posture. Please consider that participants were asked to perform the very same task in both experiments, i.e., to judge spatial relations from the co-experimenter’s point of view.

The participant’s arm posture (uncrossed vs. crossed) and the co-experimenter’s arm posture (uncrossed vs. crossed) were manipulated to obtain four types of trials (see Figure 3a):

- *participant uncrossed/co-experimenter uncrossed*: in which both the participant and the co-experimenter adopted an uncrossed posture;
- *participant uncrossed/co-experimenter crossed*: in which the participant’s arms were uncrossed, whereas the co-experimenter adopted a crossed posture, with his arms crossed over the midline;
- *participant crossed/co-experimenter uncrossed*: in which the participant adopted a crossed posture, whereas the co-experimenter held his arms uncrossed;
- *participant crossed/co-experimenter crossed*: in which both the participant and the co-experimenter adopted a crossed posture.

Each type of trial was run in a separate block. Block order was counterbalanced across participants. Front/back and left/right questions in each block were presented in a randomized order. Moreover, in order to control for object-based facilitation, the same object arrays as those used in Experiment 1 were employed. The experiment lasted approximately 30 minutes.

#### Analysis

As for Experiment 1, trials in which participants made an error were discarded from the RT analysis (3.12%). In addition, individual trials were removed when responses occurred before 150 ms after the end of the question (3.90%) or in excess of two standard deviations of the participant’s mean reaction time (3.45%). RTs were analyzed by using a repeated measures ANOVA with the *participant arm posture* (uncrossed vs. crossed), *co-experimenter arm posture* (uncrossed vs. crossed), and *response axis* (front/back vs. left/right) as within-subjects factors. Additionally, as for Experiment 1, repeated measures ANOVAs were computed on RTs separately for left/right and front/back judgments with *participant arm posture* (uncrossed vs. crossed), *co-experimenter arm posture* (uncrossed vs. crossed), and *response* (left vs. right; front vs. back) as within-subjects factors.

### Results

The ANOVA revealed a main effect of *co-experimenter arm posture* (*F*(1, 20) = 9.573, *p* = .006, η^2^ = .324), with RTs being slower when the co-experimenter adopted a crossed posture (*M* = 523,3 ms) than when he adopted an uncrossed posture (*M* = 465,5 ms). Furthermore, there was a significant main effect of *response axis* (*F*(1, 20) = 11.267, *p* = .003, η^2^ = .360), reflecting slower RTs for judgments of left/right relations (*M* = 517,8 ms) than for judgments of front/back relations (*M* = 471 ms). The effect of *participant arm posture* was not significant (*F*(1, 20) = .368, *p* = .551, η^2^ = .018), indicating that participants were equally slow when adopting an uncrossed (*M* = 489,9 ms) or crossed (*M* = 498,9 ms) posture. No interaction effect resulted to be significant (all *Fs*<.828, *p>*.374).

#### Analysis of RTs for left/right judgments

**Figure 4:**
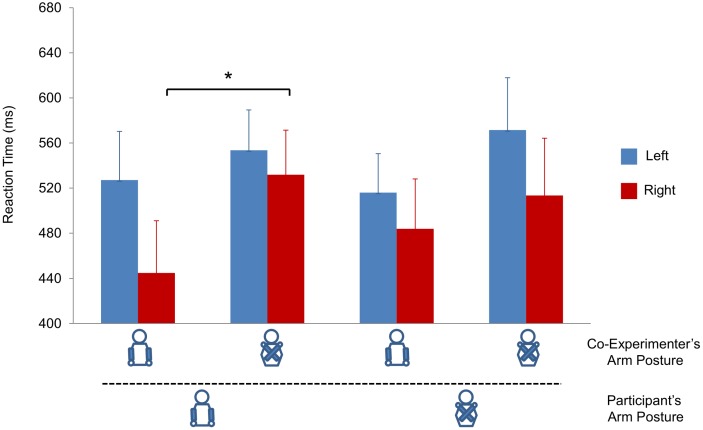
Results of Experiment 2 for left vs. right judgments. Graphical representation of the three-way interaction between *Participant Arm Posture* (uncrossed vs. crossed), *Co-experimenter Arm Posture* (uncrossed vs. crossed), and *Response* (left vs. right). Bars represent standard errors of the means. Asterisks indicate significance for the main contrasts of interest (*p*<05).

The ANOVA showed a main effect of *co-experimenter arm posture* (*F*(1, 20) = 5.438, *p* = .030, η^2^ = .214), with participants responding more slowly when the co-experimenter adopted a crossed posture (*M* = 542,6 ms) compared to an uncrossed posture (*M* = 493 ms). Furthermore, a main effect of *response* was found (*F*(1, 20) = 10.194, *p* = .005, η^2^ = .338), with right responses (*M* = 493,5 ms) being faster compared to left responses (*M* = 542,1 ms). The main effect of *participant arm posture* was not significant (*F*(1, 20) = .147, *p* = .706, η^2^ = .007) and there were no significant two-way interactions (all *Fs<*.706, *p>*.411), but there was a significant three-way interaction between *participant arm posture*, *co-experimenter arm posture,* and *response* (*F*(1, 20) = 5.374, *p* = .031, η^2^ = .212). The three-way interaction was followed up with a two (*co-experimenter arm posture*) by two (*response*) ANOVA for each *participant arm posture* (uncrossed vs. crossed) and, where significant interactions were found, two tailed t-tests were applied. For the *participant crossed arm position*, there was no significant interaction between *co-experimenter arm posture* and *response* (*F*(1, 20) = 1.131, *p* = .300, η^2^ = .054). For the *participant uncrossed arm position*, the interaction *co-experimenter arm posture* by *response* approached significance (*F*(1, 20) = 3.890, *p* = .063, η^2^ = .163). When exploring the interaction effects, it emerged that right responses were faster when the co-experimenter held his arms uncrossed than when he held his arms crossed (*M* = 444,8 vs. *M* = 531,9 ms, respectively; *t*(20) = −2.573, *p* = .018). In contrast, the co-experimenter’s arm posture did not significantly affect reaction times for left responses (*M* = 527,1 vs. *M* = 553,6 ms; *t*(20)* = −*1.103, *p* = .283; see Figure 4).

#### Analysis of RTs for front/back judgments

The ANOVA showed a main effect of *co-experimenter arm posture* (*F*(1, 20) = 6.855, *p* = .016, η^2^ = .255), reflecting slower RTs when the co-experimenter’s arms were crossed (*M* = 495,4 ms) than when they were uncrossed (*M* = 445,8 ms). The main effect of *participant arm posture* (*F*(1, 20) = .324, *p* = .575, η^2^ = .016) and the main effect of *response* did not reach significance (*F*(1, 20) = 1.837, *p* = .190, η^2^ = .084). Moreover, no interaction effect was found to be significant (all *Fs<*3.243, *p*>.087).

This pattern of results suggests that participants were generally slower to respond when the co-experimenter adopted a crossed posture than when he adopted an uncrossed posture. In addition to this general effect, observing a person holding his arms crossed specifically affected left/right judgments. Breaking down the three-way interaction indicated that right responses when the co-experimenter held his arms uncrossed were faster than right responses when he held his arms crossed. This was not the case for left responses. This pattern of results was observed when the participant’s arms were uncrossed, but not when they were crossed, suggesting that congruency between one’s own body posture and that of the other person may modulate the effect [20]. As far as front/back judgments are concerned, the only significant effect was found for the *co-experimenter arm posture* factor, indicating that both front and back responses were slower when the co-experimenter’s arms were crossed than when they were uncrossed.

## Experiment 3

The findings of Experiment 2 can be explained by the assumption that the encoding of spatial relations from another person’s perspective is structured relatively to the other person’s arm posture. If this is correct, then no specific effect of arm posture manipulation on left-right relations should be observed when taking the perspective of a person holding his arms uncrossed with his hands on his shoulders. This might occur because in this situation, the relative position of the hands, although unusual, corresponds to the side of the body with which the hands are connected. To test this prediction, in Experiment 3 we asked participants to perform the spatial judgments task from the perspective of a person holding his arms uncrossed with his hands on his shoulders. We expected response latencies to become overall longer reflecting the unusual posture, but that no specific effect would be observed on left/right judgments.

### Methods

#### Participants

Twenty undergraduate students (10 females; mean age: 20.52±2.52, range 19–27 years) from the University of Turin volunteered to take part in the experiment. All had normal or corrected-to-normal vision, were right handed, and naïve with respect to the purpose of the study. None of the participants who took part in Experiments 1 and 2 participated in Experiment 3.

#### Materials and apparatus

The materials and apparatus were the same as in Experiment 2, with the sole exception that the participants and the co-experimenter were asked to hold their arms either uncrossed with their hands on the armrest of the chair (from here on termed as “usual” posture since it was the very same as that of the previous experiments) or on their shoulders so that the left hand was placed on the left shoulder and the right hand on the right shoulder (from here on termed as “unusual” posture in opposition to usual posture).

### Design and Procedure

The *participant arm posture* (usual vs. unusual posture) and the *co-experimenter arm posture* (usual vs. unusual posture) were manipulated to obtain four types of trials, run in separate blocks (see Figure 3b):

- *participant usual/co-experimenter usual posture:* in which both the participant and the co-experimenter held their arms uncrossed with their hands on the armrest;
- *participant usual/co-experimenter unusual posture*: in which the participant held his/her arms uncrossed and his/her hands on the armrest, whereas the co-experimenter’s arms were bent at the elbows, uncrossed and his hands were on his shoulders;
- *participant unusual/co-experimenter usual posture*: in which the participant’s arms were uncrossed and his/her hands were on his/her shoulders, whereas the co-experimenter’s arms were uncrossed and on the armrest;
- *participant unusual/co-experimenter unusual posture*: in which both the participant and the co-experimenter held their arms uncrossed with their hands on their shoulders.

#### Analysis

Trials in which participants made an error were discarded from the analysis (3.34%). In addition, individual trials were discarded if responses were made less than 150 ms after the end of the question (4.09%) or in excess of two standard deviations of the participant’s mean reaction time (3.31%). RTs were analyzed using a repeated measures ANOVA with *participant arm posture* (usual vs. unusual posture), *co-experimenter arm posture* (usual vs. unusual posture), and *response axis* (front/back vs. left/right) as within-subjects factors. As for Experiment 2, repeated measures ANOVAs were computed on RTs separately for left-right and front-back judgments with *participant arm posture* (usual vs. unusual posture), *co-experimenter arm posture* (usual vs. unusual posture) and *response* (left vs. right; front vs. back) as within-subjects factors.

### Results

The ANOVA revealed a main effect of *co-experimenter arm posture* (*F*(1, 19) = 6.920, *p* = .016, η^2^ = .267), reflecting slower RTs in the unusual (*M* = 541 ms) than in the usual posture (M = 490,6 ms). The effects of *participant arm posture* (*F*(1, 19) = .003, *p* = .960, η^2^ = .000) and *response axis* were not significant (*F*(1, 19) = .191, *p* = .667, η^2^ = .010). Moreover, no interaction effect was revealed (all *Fs<*2.183, *p*>.156).

#### Analysis of RTs for left/right judgments

The ANOVA showed a main effect of *response* (*F*(1, 19) = 10.518, *p* = .004, η^2^ = .356), reflecting slower RTs for judgments of left relations (*M* = 512,6 ms) than for judgments of right relations (*M* = 485 ms). The main effect of *participant arm posture* (F(1,19) = .032, *p* = .860, η^2^ = .002) and of *co-experimenter arm posture* (F(1,19) = 2.929, *p = *.103, η^2^ = .134) were not significant. Furthermore, no interaction effect was revealed (all *Fs<*.777, *p>.389*).

#### Analysis of RTs for front/back judgments

The ANOVA showed a main effect of the *co-experimenter arm posture* (*F*(1, 19) = 6.684, *p* = .018, η^2^ = .260) with slower RTs when the co-experimenter held his hands on his shoulders (*M* = 565 ms) compared to when he held them on the armrest (*M* = 496,6 ms). The main effect of *participant arm posture* (*F*(1,19) = .004, *p* = .953, η^2^ = .000) and of *response* (*F*(1,19) = 1.940, *p* = .180, η^2^ = .093) were not significant. Furthermore, no interaction effect was revealed (all *Fs<*3.008, *p>.*099).

As predicted, these findings clearly indicate a general increase in response latencies for both left/right and back/front judgments. Critically, right responses were faster than left responses regardless of the co-experimenter’s hand posture, indicating that the unusual posture did not interact with left-right judgments. For front-back judgments, the pattern of results was similar to Experiment 2, with slower front/back responses when the co-experimenter held his arms on his shoulders.

## General Discussion

Previous evidence suggests that people taking another person’s perspective remap objects and locations relatively to the other person’s body. But how is the other person’s body represented during these tasks?

In order to address this issue, in the present study we systematically manipulated arm position in a task in which spatial judgments were made from the perspective of the participant or from that of a co-experimenter. Our results demonstrate that crossing the arms over the midline had a specific impact on spatial judgments from a first-person perspective (Experiment 1). Critically, similar effects on spatial judgments from a third-person perspective were observed when the participants adopted the perspective of a person holding his arms crossed (Experiment 2), but not when the other person’s arms were held in an unusual, uncrossed arm posture (Experiment 3). These findings might be taken to suggest that the other person’s body plays an important role in structuring the altercentric representation of space. Accumulating evidence indicates that within an egocentric frame of reference, locations are coded neither in relation to a bodily point, nor the other person’s body as a whole, but to specific body parts, such as the face, the hands, and the arms [1], [2], [3]. Our data raise the intriguing possibility that altercentric space may be structured in a similar body-part-centered manner. It may be objected that when taking the co-experimenter’s perspective, participants did not remap spatial locations with reference to the other person’s arm posture, but simply translocated the origin of the egocentric coordinate system to the other person’s bodily position [41]. If this were the case, however, effects on left/right spatial judgments from a third-person perspective should have been observed when participants held their arms crossed, but not when the co-experimenter held his arm crossed. Future studies where the position of the head or the legs is manipulated might help to clarify whether similar principles apply to other body parts.

**Figure 5:**
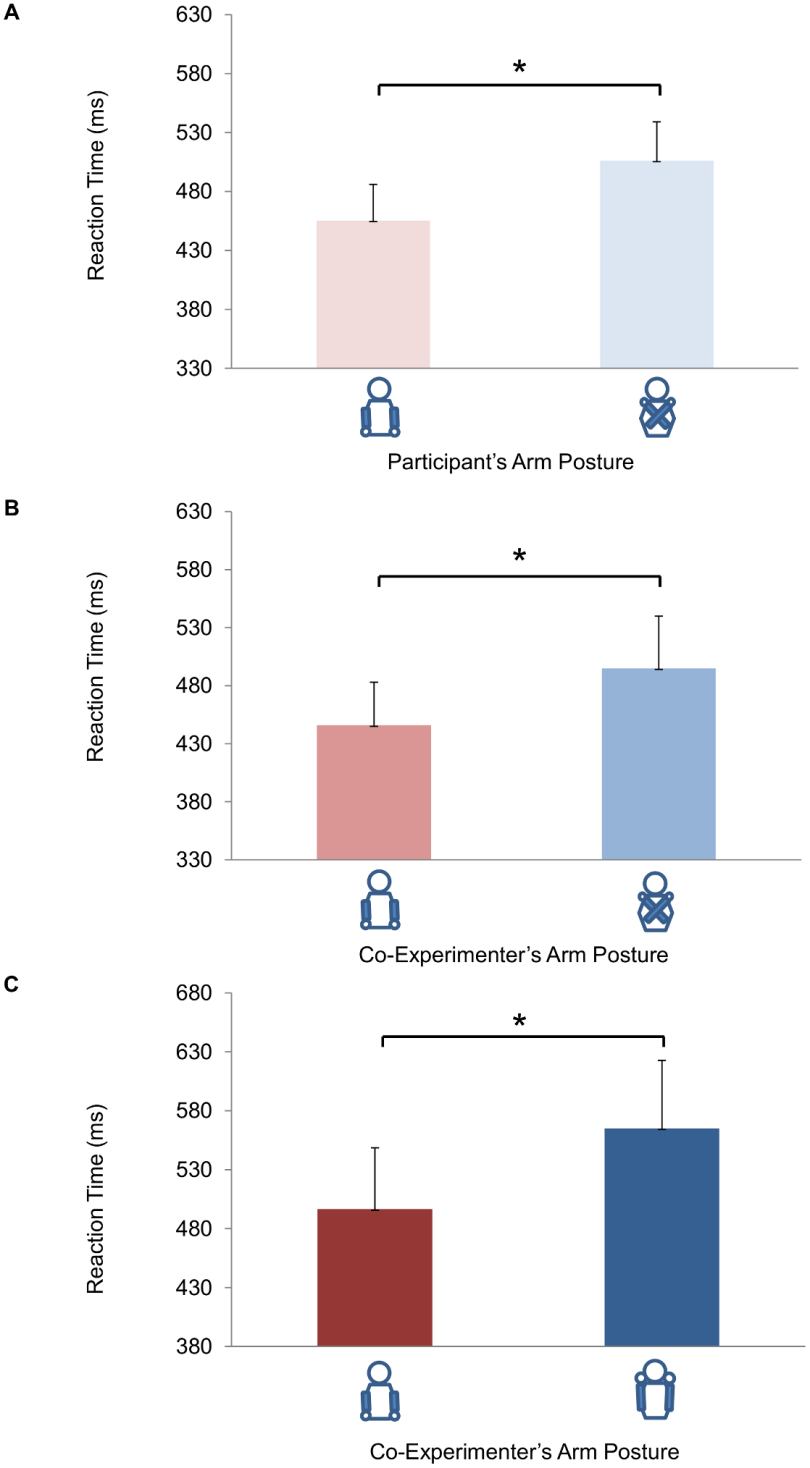
Effects of experimental manipulation on front/back judgments in the three experiments. Main effect of *Participant Arm Posture* in Experiment 1 (Panel A) and main effect of *Co-experimenter Arm Posture* in Experiments 2 and 3 (Panel B and C, respectively) on Reaction Times. Bars represent standard errors of the means. Asterisks indicate significance for the main contrasts of interest (*p*<05). Please note that all these main effects refer to the very same hand-object distance.

A second implication of our results is that the altercentric frame of reference cannot be reduced to an object-centered or allocentric frame of reference, which happens to be centered on the body of another person. Unlike object-centered or allocentric frames of reference, the egocentric frame of reference is construed out of the bodily axes “which are immediately used by the subject in the direction of action” [42]. Two prominent anatomical axes, front/back and left/right, are natural reference axes for organizing horizontal space surrounding the body [43], [44]. Our findings suggest that the horizontal plane surrounding *other bodies* may be organized along the same axes and that arm crossing by the other person may specifically perturb spatial representation along the left-right axis. The fact that the co-experimenter’s adoption of a crossed-arm posture also influenced front/back judgments does not detract from the predicted specific effect and may indeed reflect the influence of arm crossing on the front-back axis. To explain, in our setting, crossing the arms over the midline did not only alter the spatial correspondence of the hands with left/right locations, but also affected the antero-posterior dimension. When the participants crossed their arms, they put their hands on their shoulders. Under these circumstances the distance between the participants’ hands and the objects in the array was greater than when they placed their hands on the armrest and no crossing was requested. It is thus possible that participants were slower to judge front/back relations when the co-experimenter’s arms were crossed because in this situation the co-experimenter’s hands were farther away from the objects to be judged. While further studies in which the position of the hand on the antero-posterior axis is manipulated are certainly needed to clarify the effect of hand proximity, two findings support the suggestion that the distance between the hands and the objects might represent the variable leading to the reported effect. First, in Experiment 1 we found that front/back judgments from a first-person perspective were similarly affected when the participant assumed a crossed posture (see Figure 5a). Second, a similar effect on front/back judgments was observed in Experiment 3, in which the spatial judgments task was performed from the perspective of a person holding his arms uncrossed with his hands on his shoulders. Since in this situation the distance between the hands and the objects was the same as in the crossing situation, this may explain why in Experiments 2 and 3 similar effects were observed for front/back judgments, but not for left/right judgments (see Figure 5b and 5c). This result suggests that the crossing manipulation and the arm-on-the shoulder manipulation had similar effects on the front-back axis, but different effects on the left-right axis.

First-person perspective has been described as ‘bodily’ in that ‘‘the body is the subject’s point of view on the world. One’s own location, which determines what one can perceive, is the location of one’s body, and perceived objects are perceived as standing in spatial relations to one’s body’’ [45]. In accordance with the results of previous research [46], [47], our findings indicate that third-person perspective is also bodily, in that it is anchored on the other person’s body and is specifically influenced by the other person’s body posture. It has been proposed that a common body scheme is used to represent both one’s own body and the body of others [48]. These findings suggest that the commonality is not only between the representation of one’s own body and the body of others, but extends to the interpersonal mapping of egocentric and altercentric space.
